# Defect-insensitive bound states in the continuum in antisymmetric trapezoid metasurfaces in the visible range

**DOI:** 10.1515/nanoph-2025-0406

**Published:** 2025-10-07

**Authors:** Chenyu Liao, Lidan Zhou, Baohua Wen, Xiangyi Ye, Hongjiang Zhu, Ji Yang, Guohua Li, Zhangkai Zhou, Jianhua Zhou, Jingxuan Cai

**Affiliations:** School of Biomedical Engineering, 26469 Shenzhen Campus of Sun Yat-sen University, Shenzhen, 518107, China; School of Biomedical Engineering, Sun Yat-sen University, Guangzhou, 510275, China; State Key Laboratory of Optoelectronic Materials and Technologies, School of Electronics and Information Technology, Sun Yat-sen University, Guangzhou, 510006, China; Quantum Science Center of Guangdong-Hong Kong-Macao Greater Bay Area, Shenzhen, 440303, China

**Keywords:** nanophononics, symmetry-protected BIC, defect-insensitive, metasurface

## Abstract

Symmetry-protected bound states in the continuum (SP-BIC) enable flexible tuning of the wavelengths and linewidths of high-Q resonances, showing great potential in high-performance photonic devices. However, the implementation of SP-BIC at shorter wavelengths, such as in the visible range, requires precise control of smaller feature sizes, which imposes stringent fabrication requirements. This trade-off between geometric accuracy and manufacturing complexity limits the practical application of SP-BIC metasurfaces. This work presents a defect-insensitive design strategy for SP-BIC metasurfaces by constructing position-detuned arrays composed of periodically arranged antisymmetric trapezoidal unit cells. Theoretical studies show that under x-polarized incidence, the dominant resonance mode is a magnetic dipole, with electric fields strongly enhanced in the inter-pillar gap rather than inside the structures body. This field distribution provides high robustness against geometric deformation. The geometric dependence of this mode under x-polarized light is approximately 1/10 to 1/20 that of the electric dipole-dominated mode under y-polarized light, and the design is demonstrating strong tolerance to typical fabrication defects encountered in nanofabrication. Experimental validation is being conducted using metasurfaces fabricated with intentional defects. Overall, this work is offering a practical, fabrication-tolerant approach to realizing high-performance dielectric BIC metasurfaces.

## 1 Introduction

In recent years, bound states in the continuum (BIC) have attracted widespread attention due to their unique physical characteristics and superior performance metrics [1], [2]. The concept of BIC originates from non-radiating states in quantum mechanics [3], as a special class of electromagnetic eigenstates [4], [5], [6], BICs can support fully localized resonant modes embedded within the radiation continuum [7], theoretically enabling infinite quality-factor (Q-factor) and perfect field confinement. These properties endow BIC with tremendous application potential in high-performance photonic devices [8], including narrowband filters [9], low-threshold lasers [10], [11], efficient nonlinear optical converters [12], [13], and ultrasensitive biosensors [14], [15]. Depending on the formation mechanisms, BIC are generally categorized into three types [2]: symmetry-protected BIC [7], accidental BIC [16], and topological BIC [17]. Among them, SP-BICs suppress radiative losses by exploiting structural symmetry, offering advantages such as ease of control and high design flexibility, and have thus become a focal point of experimental research [18]. However, there are also significant limitations in the symmetry-protection mechanism, particularly the high relativity of the resonant modes to structural geometry [19], even nanometer-scale deviations in the geometry may lead to notable resonance shifts or spectral distortion. For example, studies have shown that even a geometric deviation with a standard deviation of *σ* = 0.9 nm during fabrication can lead to a significant degradation in the Q-factor of certain BIC designs. Specifically, the Q-factor of a split-ring design drops from 200 to 91, a reduction of 54.5 % [20].

This challenge is especially pronounced in shorter wavelength regimes (visible or ultraviolet range), where achieving the desired spectral response often requires structural features to be controlled within tens of nanometers. Such stringent demands place considerable pressure on nanofabrication techniques. Although electron beam lithography (EBL) theoretically enables resolutions as fine as 2–5 nm [21], [22], various fabrication imperfections are inevitably introduced during practical processing due to factors such as material roughness, development nonuniformity, and resist-related artifacts [23], [24]. As systematic errors of nanofabrication are consistent, the deviation of fabricated structures becomes a significant problem when designing BIC structures aim to working in shorter wavelengths, since fabrication errors have a greater impact on structures with smaller feature sizes. Hence, the high geometric dependence of SP-BIC structures and inevitable fabrication imperfection severely constrain the performance and reliability of SP-BIC-based devices in practical applications, especially in areas such as narrowband filtering with stringent frequency selectivity, nonlinear optical conversion requiring precise mode matching, and biosensing that demands long-term spectral stability and consistency. Meanwhile, the devices operating at shorter wavelength (i.e. visible range) offer inherent advantages for imaging-based sensing applications, as the optical responses can be directly recorded by silicon photodiode-based devices or observed by the naked eye [25]. Therefore, improving the tolerance of SP-BICs to structural imperfections or developing novel architectures with inherent robustness to fabrication errors is essential for realizing practical, cost-effective, and high-performance devices in the visible regime. Such defect-insensitive BIC designs are also compatible with template-based replication techniques, including nanoimprint lithography and mask photolithography, thereby facilitating the mass production of BIC-based photonic devices [26].

Here, we present a new design of all-dielectric BIC metasurface, which is composed of a periodic array of asymmetric trapezoidal silicon pillars on a quartz substrate. By introducing lateral displacement to break structural symmetry, the ideal BIC is transformed into a radiative quasi-BIC (q-BIC) mode. Simulation reveals that under x-polarized light, the q-BIC resonance is dominated by a magnetic dipole (MD) mode. We systematically analyzed the influence of geometric parameters on the resonance characteristics and found that the MD-dominated q-BIC peak exhibits significantly lower dependence on structural dimensions, with relativity merely 1/10 to 1/20 that of the electric dipole (ED)-dominated mode. Furthermore, we investigated common imperfections in nanofabrication, and the results indicate that the MD-resonant peak remains spectrally stable with minimal shift or distortion under these defects, demonstrating exceptional tolerance and defect insensitivity. Experimentally, we fabricated the designed metasurface using EBL and characterized both its geometry and optical responses, further confirming the design’s robustness to fabrication-induced deviations. This work provides a practical and defect-insensitive design strategy for BIC metasurfaces in the visible range, with promising potential for high-resolution imaging, optical sensing, and integrated photonic applications.

## 2 Results and discussion

**Figure 1:**
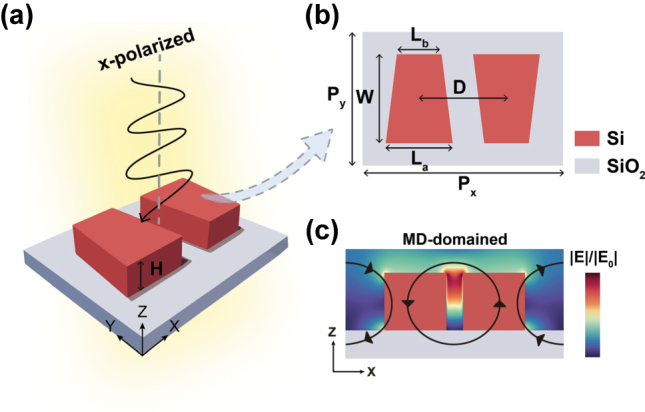
Antisymmetric trapezoid silicon pillar BIC metasurface. (a) Schematic illustration of the proposed antisymmetric trapezoid silicon metasurface supporting BIC; (b) top-view layout and structural parameters of a single unit cell; (c) schematic diagram of the simulated electric field distribution in the x-z plane at *y* = 0 nm under X-polarization.

Figure 1a illustrates the schematic of the proposed all-dielectric metasurface unit cell, which consists of a pair of antisymmetric silicon trapezoidal pillars. As shown in the top-view layout (Figure 1b), the trapezoidal pillars with identical height (*H* = 100 nm) exhibit central symmetry with respect to the z-axis. *P*_*x*_ and *P*_*y*_ represent the periodicities of the unit cell along the x- and y-directions, respectively. The geometric parameters of the trapezoids include the bottom length *L*_*a*_, top length *L*_*b*_, width *W* of the trapezoids and the inter spacing between the two trapezoids *D*. We performed numerical simulations using the finite-difference time-domain (FDTD) method to investigate the optical response of the designed structure. To quantitatively characterize the degree of translational symmetry breaking that enables the excitation of BIC, we define an asymmetry parameter as $\alpha =\left|2D-P_{x}\right|/P_{x}$ . In the simulations, the metasurface is assumed to be surrounded by air (background refractive index *n* = 1). Periodic boundary conditions are applied along both x and y axes, while a perfectly matched layer (PML) is employed along the z-direction to absorb outgoing wave. We investigated the optical properties of the antisymmetric trapezoid metasurface under x-polarized light. Figure 1c shows a schematic diagram of the simulated electric field distribution in the x-z plane under X-polarization. The black arrows represent the current direction, indicative of an MD-dominated resonance.

**Figure 2:**
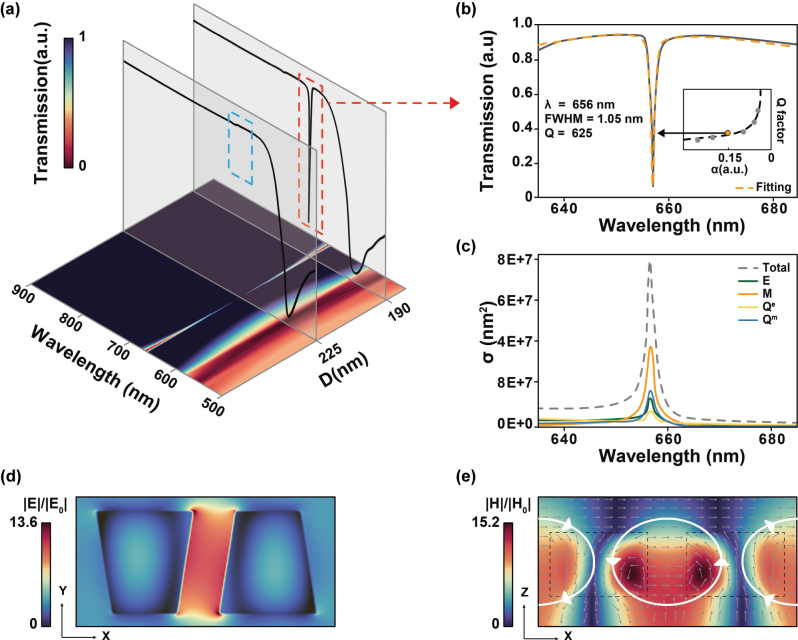
q-BIC resonance mechanism in x-polarized light. (a) Simulated transmission spectral distribution. The horizontal axis indicates the antisymmetric trapezoidal structure spacing and the wavelength, respectively. The color bar indicates transmittance; (b) transmission spectrum at asymmetry factor *α* = 0.15, along with the corresponding Fano fitting curve. The inset shows the simulated Q-factor as a function of α; (c) multipole decomposition of the corresponding resonance, showing the scattering cross sections (*σ*) of different modes (ED: *E*, MD:*M*, EQ: *Q* ^*e*^and MQ: *Q* ^*m*^); (d) electric field distribution in the x-y plane at z = 50 nm, illustrating field enhancement in the gap region; (e) magnetic field distribution in the x-z plane at y = 0 nm. Grey arrows indicate the displacement current density, while white arrows represent the direction of current vectors.

For further analysis, the unit cell was configured with periodicities *P*_*x*_ = 450 nm and *P*_*y*_ = 300 nm, and trapezoid dimensions set to *L*_*a*_ = 150 nm, *L*_*b*_ = 100 nm, and *W* = 200 nm. As shown in Figure 2a, when the asymmetry factor *α* = 0 (corresponding to *D* = 225 nm), the transmission spectrum exhibits an ideal BIC with a theoretically infinite quality factor, manifesting as an ultranarrow resonance peak with no radiative loss into free space. By breaking symmetry through varying *D* (*α* > 0), while keeping other structural parameters unchanged, we observed a progressive broadening of the resonance linewidth with increasing asymmetry. SP-BIC are inherently linked to Fano resonances, and the resulting q-BIC modes typically exhibit asymmetric Fano line shapes in the spectral response [1]. The Fano-Anderson model provides a theoretical framework for describing the coupling and interference between discrete resonant states and the surrounding continuum, offering deeper insight into the scattering dynamics of q-BIC [27].

Thus, we performed fitting using the classical Fano equation [28]:

$$\begin{array}{c} T\left(\omega \right)=T_{0}+A_{0}\frac{{\left[q+2\left(\omega -\omega_{0}\right)/\tau \right]}^{2}}{1+{\left[2\left(\omega -\omega_{0}\right)/\tau \right]}^{2}} \end{array}$$

where *T*_0_ is the transmission offset, *A*_0_ is the coupling coefficient between the continuous and discrete states, *τ* is the resonance linewidth, *q* is the Fano asymmetry parameter, *ω* is the resonance frequency. In *ω*_0_ = 2*πc*/*λ*, *c* is the vacuum speed of light, *λ* is the resonant wavelength. Then, based on the fitted parameters, the Q-factor can be calculated using:

$$\begin{array}{c} Q_{\tau }=\frac{\omega_{0}}{\tau } \end{array}$$

Hence, the resonance peak wavelength at an asymmetry factor of *α* = 0.15 (corresponding to *D* = 190 nm) is determined to be 656 nm, and the full width at half maximum (FWHM) is 1.05 nm, corresponding to a Q-factor of approximately 625.

The inset of Figure 2b shows the relationship between the Q-factor and the asymmetry factor, *α*. The fitting process is based on the linear relationship between the Q-factor and *α*.

In order to better understand the excitation mechanism of this work, the scattering power of the q-BIC resonance peak excited under this structural parameter is further analyzed for different multipoles in Cartesian coordinate system, including ED: ***p***, MD: ***m***, electric quadrupole (EQ): *Q* ^*e*^and magnetic quadrupole (MQ): *Q* ^*m*^, with four multipole moments denoted as [29], [30]:

$$\begin{array}{c} p_{\alpha }\approx -\frac{1}{iw}\int J_{\alpha }d^{3}r \end{array}$$

$$\begin{array}{c} m_{\alpha }\approx \frac{1}{2}\int {\left(r\times J\right)}_{\alpha }d^{3}r \end{array}$$

$$\begin{array}{c} Q_{\alpha \beta }^{e}\approx -\frac{1}{iw}\int \left[3\left(r_{\beta }J_{\alpha }+r_{\alpha }J_{\beta }\right)-2\left(r\cdot J\right)\delta_{\alpha \beta }\right]d^{3}r \end{array}$$

$$\begin{array}{c} Q_{\alpha \beta }^{m}\approx \int \left[r_{\alpha }{\left(r\times J\right)}_{\beta }+r_{\beta }{\left(r\times J\right)}_{\alpha }\right]d^{3}r \end{array}$$

where ***J*** is the intracell displacement current density from the electric field distribution $E\left(r\right)$ :

$$\begin{array}{c} J\left(r\right)=-i\omega \varepsilon_{0}\left(n^{2}-1\right)E\left(r\right) \end{array}$$

where *ω* is the angular frequency, *ɛ*_0_ is the permittivity of free space, *n* is the refractive index, ***r*** is displacement vector, *c* means displacement vector, *α*, *β* = *x*, *y*, *z* and *k* is wavenumber, *E*_0_ is the amplitude of the incident electric field.

Based on this, we performed a multipole decomposition analysis of the resonance peak at the resonance wavelength of 656 nm. The results are shown in Figure 2c, where the vertical axis represents the scattering cross sections (*σ*). The analysis reveals that the q-BIC resonance is predominantly governed by the MD. Figure 2d displays the normalized electromagnetic field distribution of the metasurface at this wavelength under x-polarized light. The relative lateral displacement between the two trapezoidal structures breaks the original spatial symmetry, introducing dipole moment components that enable coupling of the previously symmetry-protected mode to free space [31], thereby resulting in observable radiative leakage. The electromagnetic energy is highly confined in the gap between the two structures (Figure 2d). The strong field confinement induces oppositely directed displacement currents that circulate in a closed loop, giving rise to a magnetic dipole moment normal to the metasurface plane. Figure 2e demonstrates the magnetic field distribution, where the gray arrows represent the displacement current density, while the white arrows indicate the direction of current flow. Within each unit cell, the displacement currents exhibit clockwise circulation, whereas the neighboring unit cells display counterclockwise rotation. This head-to-tail pattern of displacement currents across adjacent elements is a distinctive signature of MD resonance. Additionally, Figure S1 in supporting information illustrates the transmission response under y-polarized excitation using the same structural parameters. The results reveal an ED-dominated resonance mode, with the electric field hotspots predominantly located inside the trapezoidal structures and along their edges.

**Figure 3:**
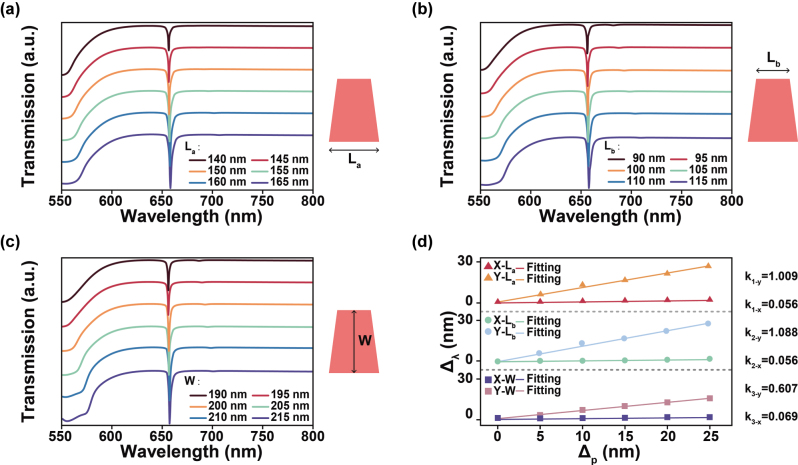
Spectral response of the antisymmetric trapezoidal structure to geometric variations and the resulting resonance wavelength shifts under x-polarized light. (a) Transmission spectra as the bottom length*L*_*a*_ of the trapezoid varies from 140 nm to 165 nm; (b) transmission spectra as the top length *L*_*b*_ of the trapezoid varies from 90 nm to 115 nm; (c) transmission spectra as the height *W* of the trapezoid varies from 190 nm to 215 nm; (d) corresponding relationship between the variation in geometric parameters (*L*_*a*_, *L*_*b*_, *W*) and the resonance peak wavelength shift under y-polarization and under x-polarization.

To investigate the relativity effects of different geometric parameters on the q-BIC resonance, we systematically examining the continuous response relationship between variations in structural dimensions and the corresponding shifts in the resonance wavelength. Figure 3 and Figure S2 present the transmission spectra under two linear polarized illumination of different axes with varying the parameters *L*_*a*_, *L*_*b*_, *W*and *P*_*x*_, along with an analysis of the correlation between these geometric changes and the resulting resonance wavelength shifts. To quantitatively characterize the relativity of the resonance wavelength *λ* to changes in structural parameters *p*, we extracted the linear fitting slope *k* = Δ*λ*/Δ*p*, which serves as an indicator of the geometric dependence of the resonance. The results show that as the geometric size and periodicity of the silicon trapezoidal structures increase, the resonance peaks generally redshift. However, the degree of the spectral response to geometric parameter variations differs significantly between two resonance modes. Figure 3d shows the q-BIC resonance dominated by MD mode, the effect of geometric changes is relatively light, with the fitted slope *k* not exceeding 0.07. In contrast, the q-BIC resonance dominated by the ED mode under y-polarized light shows much higher geometric relativity, with *k* values may exceed 1, which is 10–20 times greater than those of the MD-dominated mode. In terms of periodicity variation, the MD resonance is highly sensitive to the array period due to its dependence on circulating magnetic fields confined within the structure. Changes in periodicity significantly modulate magnetic field interference and mode coupling behavior. Conversely, the ED mode features electric field localization at the edges of the structure, making it highly sensitive to the shape and dimension of the geometry but less dependent on the periodic arrangement. As a result, MD-dominated resonances exhibit greater relativity to periodicity variations compared to ED-dominated ones. This behavior can be explained by perturbation theory, which states that the sensitivity of a resonant mode to geometric deformations depends primarily on the spatial distribution of the electric field energy [32]. Frequency shifts are more significant when the perturbation occurs in regions of high local field energy density.

The weak relationship between the resonance response and individual geometric parameters suggests that the proposed structure has low geometrical dependency, indicating inherent robustness against geometric deformation. During EBL, the achievable resolution is often limited by practical constraints such as proximity effects and resist processing variations. Optimization approaches such as proximity effect correction and dose modulation can effectively mitigate these issues, significantly improving pattern fidelity and critical dimension control for high-precision device fabrication. However, due to statistical fluctuations in electron scattering and the intrinsic chemical characteristics of resists, residual errors may still remain. The fabrication of nanostructures with smaller feature sizes and higher packing densities, which includes optimizing exposure, will therefore require more time and incur higher costs. Additionally, in template-based replication techniques such as nanoimprint lithography and mask photolithography, while array uniformity can be maintained, the structural integrity within individual unit cells is more prone to damage, introducing defects. Therefore, resonance modes with low sensitivity to geometric parameters are highly desirable, as they offer improved spectral stability and structural robustness under realistic fabrication conditions.

**Figure 4:**
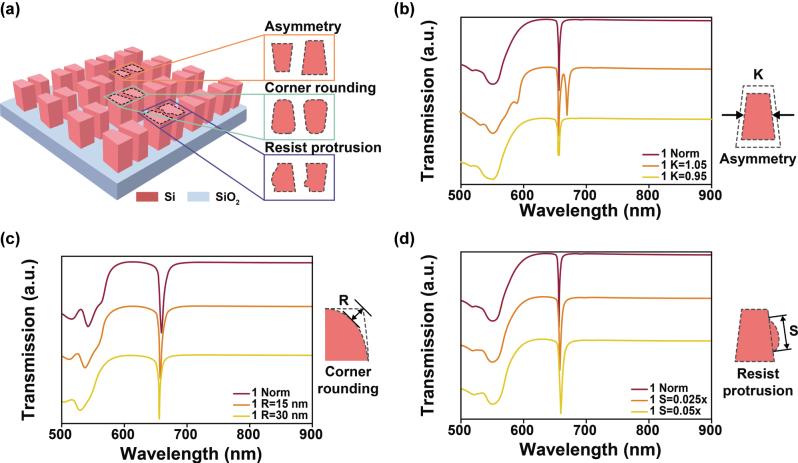
Transmission spectra of the antisymmetric trapezoid metasurface under various fabrication defects may introduced during EBL. (a) Schematic illustration of three representative defect models: asymmetry, corner rounding, and resist protrusion; (b) transmission response when the size of one trapezoid is scaled to 1.05 × or 0.95 × of the original dimension; (c) spectral variation caused by inward corner rounding of 15 nm and 30 nm; (d) transmission response when resist protrusions with volumes of 0.025 × and 0.05 × of the original structure are randomly attached to the surface of the two trapezoids.

To validate this, we introduced several common fabrication defects encountered in EBL and analyzed their impact on the spectral resonance. Figure 4a illustrates three typical types of structural imperfections: structural asymmetry between the two trapezoids, corner rounding of the originally sharp trapezoidal corners, and resist protrusion on the surface of the structures. Figure 4b presents the transmission spectra when one of the trapezoids is scaled to 1.05 and 0.95 times of its original size. Figure 4c shows the spectral response under 15 nm and 30 nm inward corner rounding. Figure 4d displays the spectral changes when random photoresist residues, with volumes of 0.025 and 0.05 times that of the original structure, are attached to the surface. The simulation results reveal that despite the presence of fabrication deviations, the primary resonance peak exhibits no significant shift or distortion across all scenarios, demonstrating the robust tolerance of the structure against typical manufacturing defects. As further confirmed by the electric field distribution analysis in Figure S3, the spatial profile of the resonance mode remains essentially unchanged under different types of defects. The localized electric field continues to concentrate within the inter-structure gap, and, critically, the dominant resonance mode remains MD.

Moreover, in Figure S4, we evaluated the impact of fabrication defects on the resonance spectra under another set of geometric parameters (*P*_*x*_ = 490 nm, *P*_*y*_ = 330 nm, *L*_*a*_ = 120 nm, *L*_*b*_ = 150 nm, *W* = 210 nm, *D* = 160 nm). The results demonstrate that even with different geometric parameters, the aforementioned typical defects did not induce significant resonance wavelength shifts. These results indicate that even when fabrication errors occur during the manufacturing process, such as dimensional deviations of individual structures reaching 5 % or more (for example, a trapezoid side length decreasing 10 nm) or the introduction of a corner rounding of about 15 nm, the resonance wavelength can still be maintained within an acceptable range relative to the ideal design. This robustness suggests that the proposed structure is not only tolerant to fabrication imperfections in high-precision processes but also potentially compatible with lower-cost, large-area fabrication techniques such as nanoimprint lithography [33], [34] or surface-directed assembly [35], [36].

**Figure 5:**
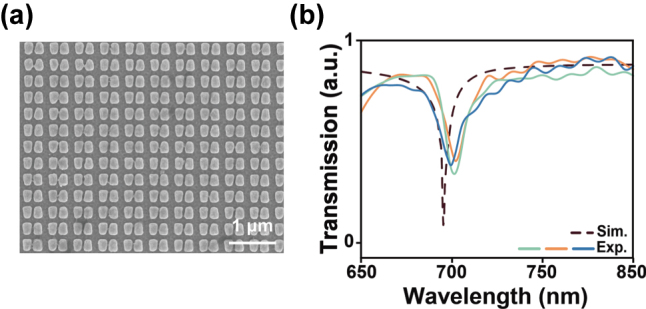
Fabrication and characterization of the all-dielectric antisymmetric trapezoidal metasurface. (a) Top-view SEM image of the fabricated metasurface; (b) simulation result and measured transmission spectra from different regions of the same metasurface batch.

Based on these parameters, we fabricated the corresponding antisymmetric trapezoid metasurface using EBL. Figure 5a presents a top-view scanning electron microscope (SEM) image of the fabricated structure. Figure 5b shows the transmission spectra measured from different areas of the same sample. Despite structural distortions in local regions, the maximum resonance peak shift was less than 5 nm, and the FWHM was 7.7 nm, validating the stability of this resonance mode under realistic fabrication conditions. In addition, we investigated the spectral response of the structure under varying ambient refractive indices. Figure S5 shows the simulated redshift of the resonance peak with increasing ambient refractive index, along with the measured transmission spectra for different glycerol concentrations. A consistent redshift trend of the resonance peak was observed with increasing refractive index. These results indicate that the proposed structure not only demonstrates strong fabrication defect tolerance, but also exhibits a stable and continuous spectral response to environmental refractive index changes, showing promising potential for functional optical device applications.

## 3 Conclusions

In summary, this work addresses the challenges posed by SP-BIC metasurfaces with small structural feature sizes, which require high structural symmetry and fabrication precision in practical applications. Simulation results reveal that under x-polarized light, the q-BIC resonance is dominated by an MD mode, with the electric field concentrated in the gap between the trapezoids. Further parameter sweeps and defect simulations demonstrate that the MD-dominated q-BIC exhibits high stability and fabrication tolerance in terms of resonance wavelength, and its geometric dependence of MD-dominated resonance is merely 1/10 to 1/20 of that of the ED-dominated resonance under y-polarization light. Experimental measurements also confirm that the resonance peak position exhibits minor shifts within an acceptable range under practical fabrication conditions. This characteristic is particularly advantageous for template-based replication techniques, such as nanoimprint lithography, where the global periodicity of the structure can be faithfully preserved even though local imperfections or damages may occur during the demolding process. Therefore, the defect-insensitive nature of our design ensures high compatibility with scalable, low-cost fabrication methods, further enhancing its potential for practical large-area applications. Therefore, this work offers a robust and defect-insensitive design strategy for BIC-based devices in the visible spectrum region.

## 4 Experimental methods

**Sample fabrication:** 100 nm poly-Si film was first deposited on a quartz substrate using plasma-enhanced chemical vapor deposition. Subsequently, a layer of PMMA positive photoresist was spin-coated on top of the silicon films. The pattern was exposed by using an electron-beam lithography system (EBPG5000Plus, Raith) operated at 100 kV. The resist was developed with methyl isobutyl ketone and isopropyl alcohol developer. Then, a thin layer of Cr was deposited via electron beam evaporation as a mask for inductively coupled plasma (Oxford Instruments) of the silicon. Finally, the Cr mask was removed, resulting in an all-dielectric silicon metasurface.

**Optical Measurements:** The transmission spectra of the samples were collected using a compact spectrometer (HR 4000, Ocean Optics) under the illumination of a halogen lamp (HL-2000, Ocean Optics).

**Refractometric Sensitivity Measurements:** Different concentrations of glycerol aqueous solutions were prepared by mixing different proportions of glycerol and deionized water, corresponding to different refractive indices. The glycerol concentrations used in the experiments were 0 % (*n* = 1.333), 5 % (*n* = 1.339), 10 % (*n* = 1.345), 15 % (*n* = 1.351), and 20 % (*n* = 1.357).

## Supplementary Material

Supplementary Material Details
