# Supplementary material for: Defect-insensitive bound states in the continuum in antisymmetric trapezoid metasurfaces in the visible range
Source: Nanophotonics. 2025 Oct 7;14(22):3681–9. doi: 10.1515/nanoph-2025-0406 (PMC12592850; doi:10.1515/nanoph-2025-0406)
Supplement: Supplementary file 1 — Supplementary Material Details [file j_nanoph-2025-0406_suppl_001.docx]

Supplementary Materials

Chenyu Liao, Lidan Zhou, Baohua Wen, Xiangyi Ye, Hongjiang Zhu, Ji Yang, Guohua Li,

Zhangkai Zhou, Jianhua Zhou*, Jingxuan Cai^[[1]](#footnote-1)^

Defect-insensitive bound states in the continuum in antisymmetric trapezoid metasurfaces in the visible range


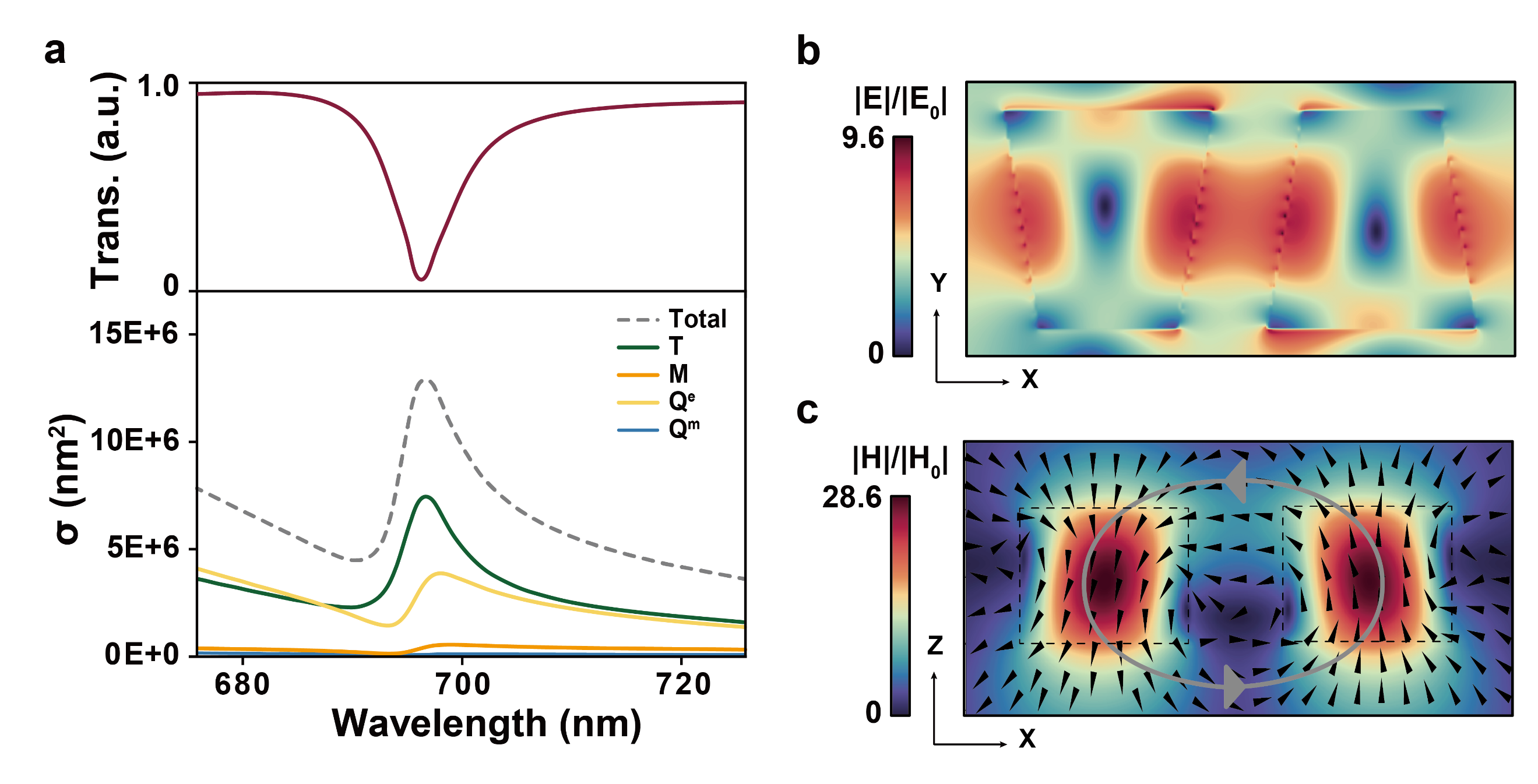


**Fig S1:** q-BIC resonance mechanism in y-polarized light. (a) Multipole decomposition of the corresponding resonance, showing the scattering cross sections (σ) of different modes (ED: $E$, MD:$M$, EQ: $Q^{e}$and MQ: $Q^{m}$). (b) Electric field distribution in the x-y plane at z = 50 nm. (c) Magnetic field distribution in the x-z plane at y = 0 nm, black cones represent magnetic field vectors and while grey arrows illustrate a counterclockwise circulation path of the magnetic field between trapezoidal elements within each unit cell.

**
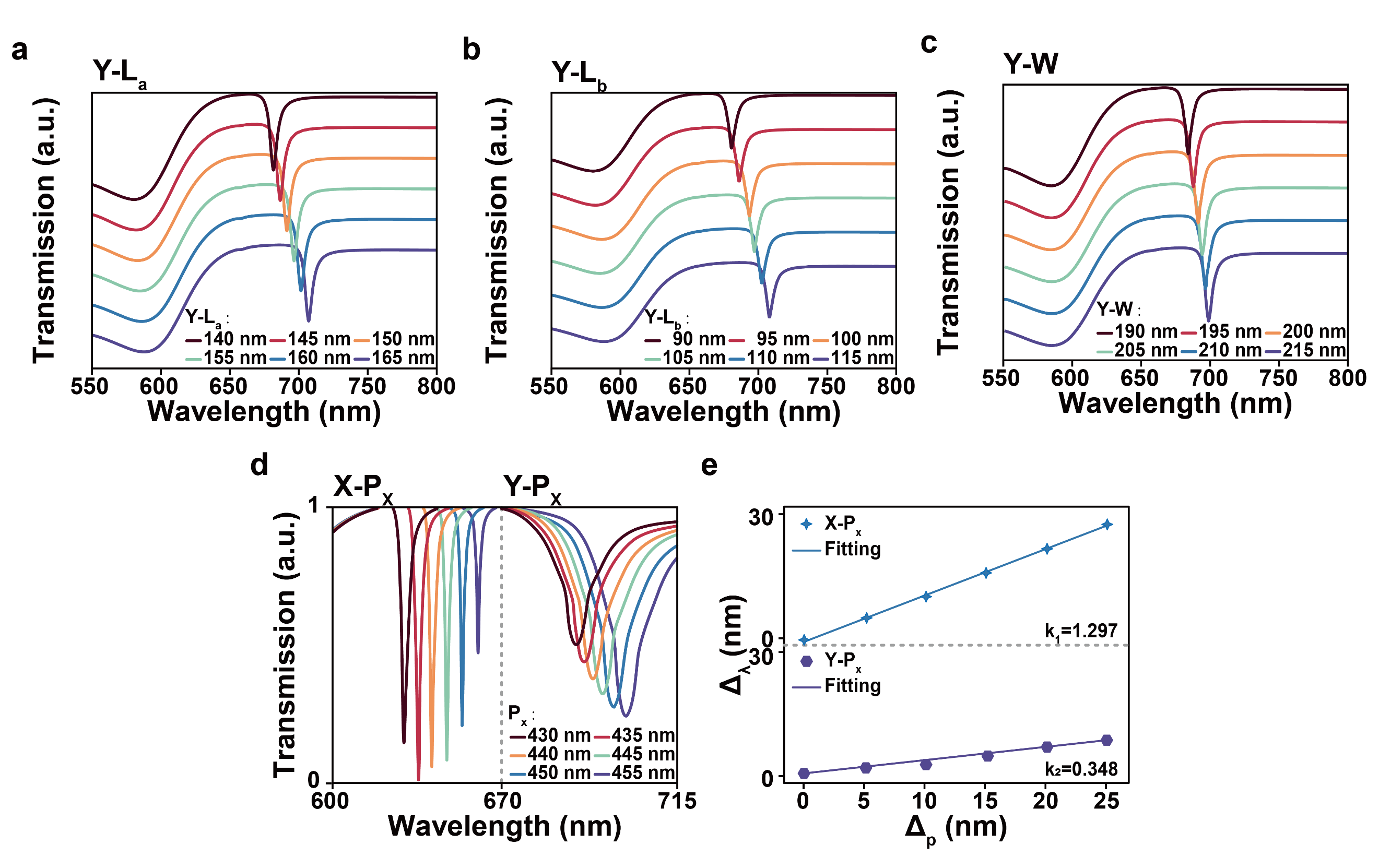
**

**Fig S2:** Spectral response of the asymmetric trapezoid structure to variations in geometric parameters; (a) Transmission spectra as the bottom length$L_{a}$​of the trapezoid varies from 140 nm to 165 nm; (b) Transmission spectra as the top length $L_{b}$​of the trapezoid varies from 90 nm to 115 nm; (c) Transmission spectra as the height $W$of the trapezoid varies from 190 nm to 215 nm; (d) Transmission spectra with continuous variation of the x-direction period $P_{x}$from 430 nm to 455 nm; (e) Correlation between the resonance wavelength shift and the variation of $P_{x}$under x-polarization and y-polarization.


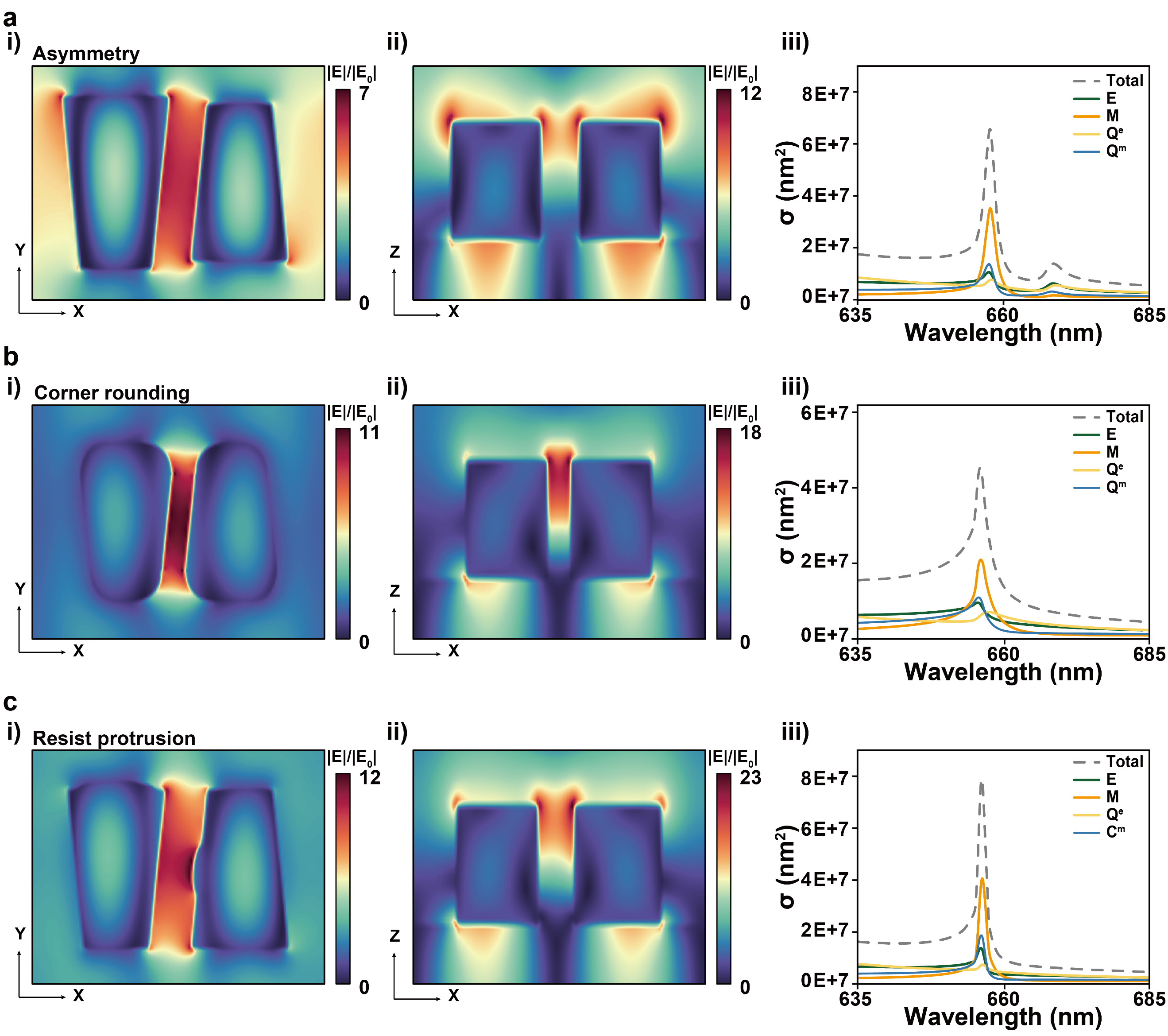


**Fig S3:** Electric field distributions and multipole decomposition of the asymmetric trapezoid structure under various nanofabrication defects. (a) Electric field distributions and multipole decomposition of the asymmetric trapezoid structure when the size of one trapezoid is scaled to 1.05× or 0.95× of the original dimension;(b) Electric field distributions and multipole decomposition of the asymmetric trapezoid structure when inward corner rounding of 15 nm and 30 nm;(c) Electric field distributions and multipole decomposition of the asymmetric trapezoid structure when resist protrusions with volumes of 0.025× and 0.05× of the original structure are randomly attached to the surface of the two trapezoids. i) Electric field distribution in the x–y plane at z = 0 nm. ii) Electric field distribution in the x–z plane at y = 0 nm. iii) Multipole decomposition of the corresponding resonance peak (ED: $E$, MD:$M$, EQ: $Q^{e}$and MQ: $Q^{m}$).


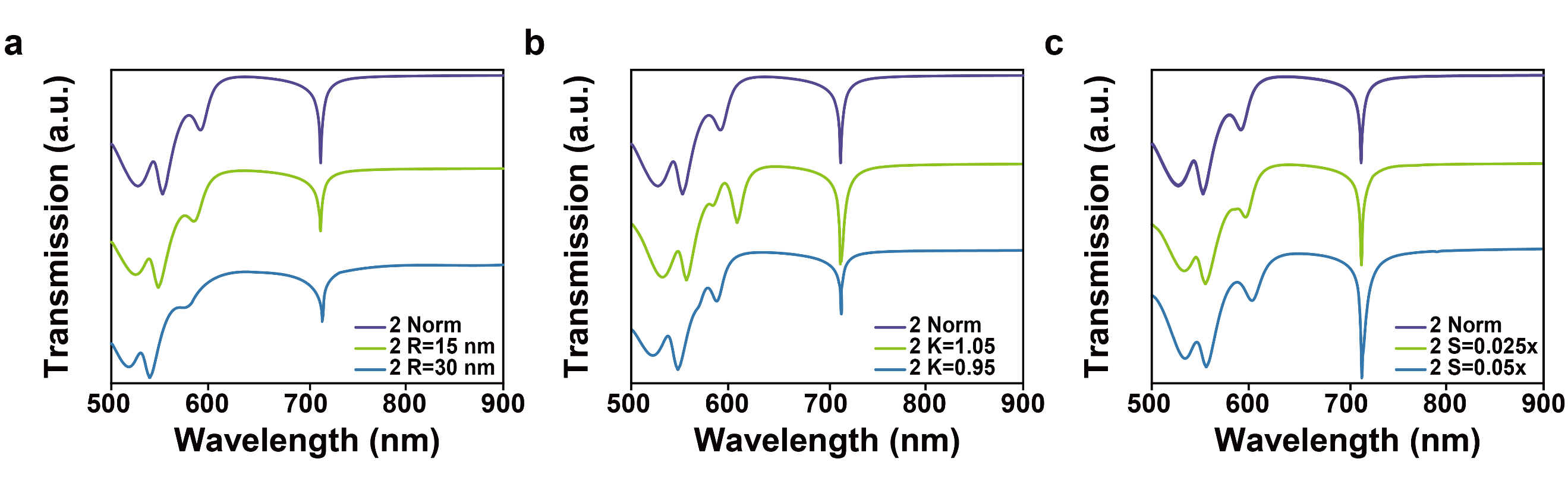


**Fig S4:** Transmission spectra of the antisymmetric trapezoid metasurface with another set of geometric parameters ($P_{x}$=490 nm, $P_{y}$=330 nm, $L_{a}$=120 nm, $L_{b}$=150 nm, $W$ =210 nm, $D$ =160 nm) under various fabrication defects may introduced during EBL.(a) Transmission response when the size of one trapezoid is scaled to 1.05× or 0.95× of the original dimension;(b) Spectral variation caused by inward corner rounding of 15 nm and 30 nm;(c) Transmission response when resist protrusions with volumes of 0.025× and 0.05× of the original structure are randomly attached to the surface of the two trapezoids.


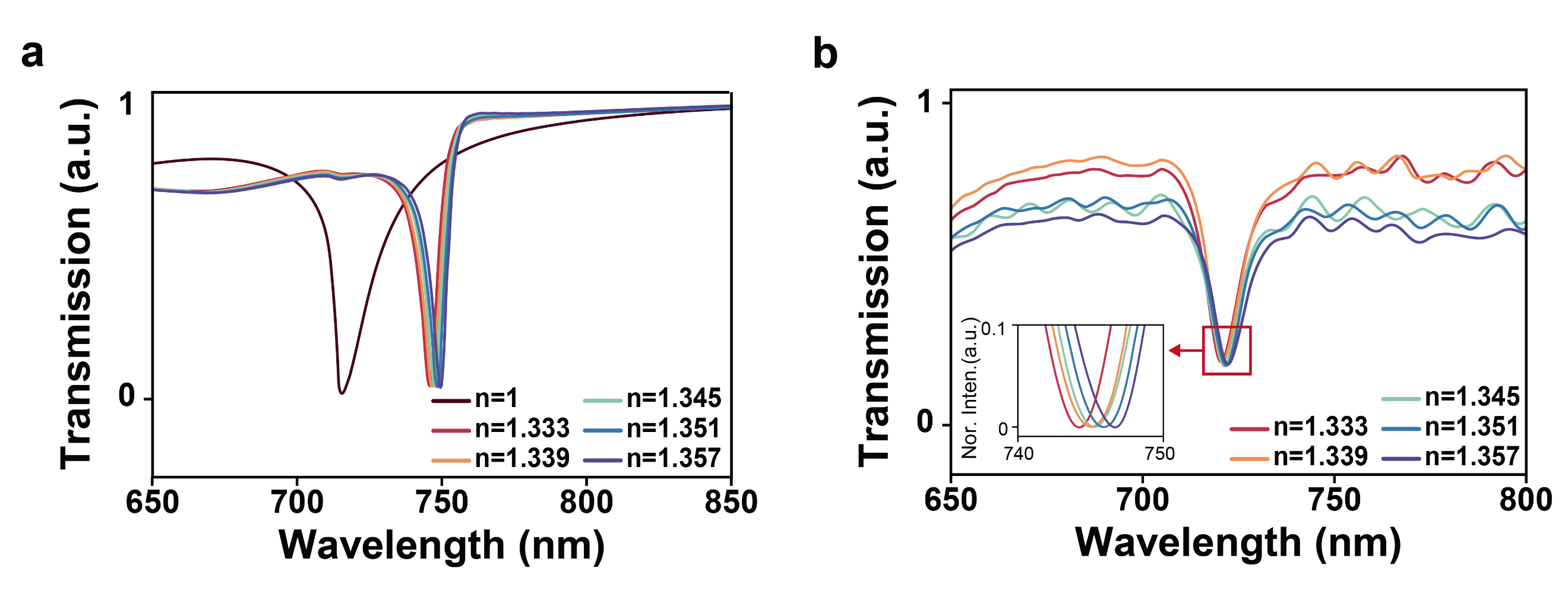


**Fig S5:** Transmission response of metasurface to environmental refractive index. (a) Simulated transmission spectra of the metasurface immersed in glycerol–water mixtures with varying refractive indices (n = 1.333-1.357); (b) Experimentally measured transmission spectra under different refractive index environments adjusted using glycerol–water mixtures (n = 1.333-1.357). The inset shows the locally magnified resonance peaks after smoothing and normalization.

1. [↑](#footnote-ref-1)
